# Finding the needle in a haystack: Single amino acids that shape viral virulence

**DOI:** 10.1080/21505594.2026.2675758

**Published:** 2026-06-03

**Authors:** David Jesse Sanchez

**Affiliations:** Pharmaceutical Sciences Department, Western University of Health Sciences, Pomona, CA, USA

The emergence of novel viral pathogens from wildlife reservoirs continues to reveal fundamental insights into viral pathogenesis [1]. Genetic variants and mutations increasingly show new ways for pathogenicity to impact different species. In a recent issue of Virulence, Yang et al. describe a highly pathogenic variant of amdoparvovirus associated with fatal respiratory disease in red pandas (*Ailurus fulgens*) [2]. Amdoparvoviruses are members of the Parvoviridae family of small, non-enveloped single-stranded DNA viruses. In the process of the genetic dissection of this pathogenesis the authors determined that a single amino acid change was sufficient to reprogram viral virulence.

## A single amino acid change in amdoparvovirus dramatically changes pathogenicity

**Figure 1.**
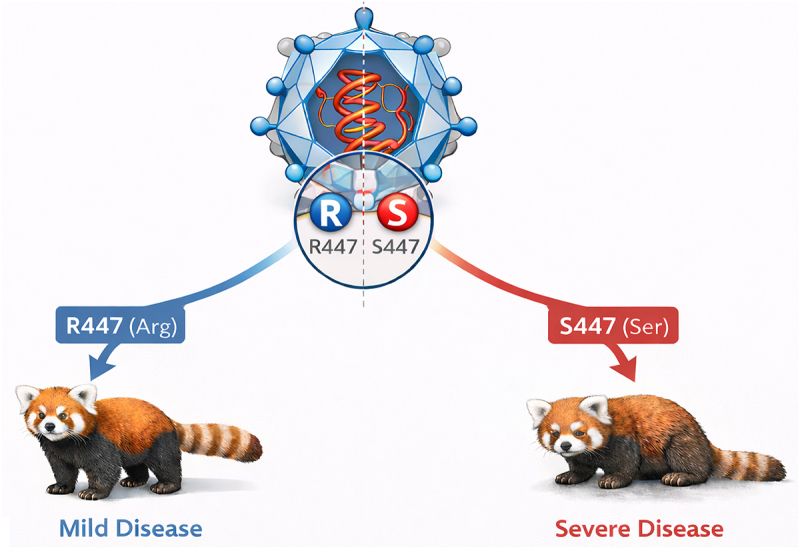
Single amino acid changes in amdoparvoviruses change disease severity.

Yang et al.’s work identifies a single amino acid residue within the VP2 capsid protein of amdoparvovirus that allows infection by this virus to have different pulmonary pathogenicity depending on the identity of the amino acid. Viruses encoding a serine at position 447 show enhanced replication, severe lung injury, and high mortality in red pandas (Summarized in Figure 1). However, viruses with an arginine at position 447 show attenuated replication and disease. This single amino acid change directly links to changes in virulence and pathogenicity showing how small genetic changes can lead to changes in virulence.

Summary of findings by Yang et al. [2]. The severity of disease caused by amdoparvoviruses of red pandas is influenced by single amino acid changes in the VP2 capsid. As illustrated, on the left side, an arginine present at VP2 position 447 leads to mild disease. In contrast, serine at VP2 position 447 is associated with severe disease, increased viral replication, acute often-fatal respiratory disease characterized by diffuse alveolar damage, and systemic inflammation. Figure created with assistance from ChatGPT by OpenAI (GPT-5 series, accessed 2026) for conceptual development and visualization refinement. The final figure was subsequently modified and finalized by the author.

The genetic basis of virulence and pathogenesis is often seen as built on complex, large changes to the viral genome. These ideas frame how we describe viral genetic changes from small changes causing mild changes to viral disease severity to large reassortments causing profound changes such as in influenza antigenic shift. Instead, the single amino acid focus of VP2-447 in amdoparvovirus reinforces a more direct view in that disease potential of a virus can be encoded in discrete structural features that determine how a virus engages with its host. The amdoparvovirus VP2 protein makes up the viral capsid and is surface exposed allowing interactions with a variety of host proteins [3]. The VP2 protein itself is not simply a structural protein of the virion but also a protein that engages with host proteins as part of viral replication influencing host disease. A single change in this protein therefore does more than alter replication as it seems to interacts with infected cells.

This interface of VP2 and host proteins it interacts with seems to be where amdoparvoviruses pathogenesis is decided. As a virion surface protein, potential VP2 interactions may define receptor engagement, cellular entry, and host range for amdoparvoviruses [4]. In other parvoviruses, capsid proteins like VP2 can facilitate antibody-dependent enhancement – like entry through Fc receptors, enabling infection of monocytes and macrophages [5]. The amdoparvovirus variants described could infect both alveolar epithelial cells and monocytic cells, linking tropism directly to lung injury and systemic inflammation. This reinforces a central point: disease severity is not just how much virus is present, but where it replicates in the host body. Infection of lung cells drives tissue damage while infection of localized immune cells amplifies inflammatory cascades further driving disease severity. The amino acids in VP2 effectively drive access to these tissue compartments, coupling cellular tropism with disease outcome.

## Single amino acid changes in other viruses can drive differences in pathogenicity

Previous studies in canine parvoviruses pointed toward multiple amino acids being the determinant of tropism in multiple species [6]. However, the importance of a single amino acid determining the pathogenicity of a virus is not unique to parvoviruses. Across virology, small genetic changes repeatedly produce large phenotypic effects when they alter host engagement. Table 1 summarizes several dramatic examples of single amino acid mutations. The influenza A virus PB2 protein is a subunit of the viral RNA-dependent RNA polymerase (RdRP) complex, but a single amino acid substitution in PB2 where glutamic acid is replaced by lysine at position 627 allows the RdRP activity to be adapted to human respiratory tracts [7]. This PB2 E627K mutation is strongly associated with avian-to-human adaptation, higher viral loads, and increased disease severity in mammalian models making it an effective biomarker [8].

**Table 1.** Single amino acid substitutions that reprogram viral tropism and virulence.

Virus	Protein	Pathogenic Mutation	Consequences
Amdoparvovirus	VP2	R447S [2]	• Increased viral replication efficiency • Enhanced lung tropism • Enhanced inflammatory response
Influenza A Virus	PB2	E627K [7,8]	• Increased polymerase activity in mammalian cells • Improved host adaptation • Higher viral loads, broader tissue tropism
Ebola virus	GP	A82V [9,10]	• Enhanced viral entry into human cells • Improved receptor interaction • Linked to higher viral titers
Chikungunya virus	E1	A226V [11,12]	• Enhanced viral fusion in mosquito • Expanded vector range • Linked to rapid spread in outbreak
Zika virus	prM	S139N [13]	• Enhanced neural progenitor tropism • Increased neurovirulence and cell death • Linked to microcephaly

Beyond polymerase adaptation, single amino acid changes frequently act at the level of viral entry, where even subtle alterations in surface proteins can reshape receptor usage and cellular tropism. In Ebola virus, the GP A82V mutation enhances viral entry into human cells by improving receptor engagement [9], and has been shown to have contributed to increased infectivity during the West African outbreak [10]. Similarly, the Chikungunya virus E1 A226V mutation alters membrane fusion efficiency in mosquito vectors [11], likely enabling expansion into the *Aedes albopictus* mosquito and facilitating spread [12]. In Zika virus, the prM S139N mutation increases neurotropism and has been linked to enhanced neural progenitor cell death and microcephaly [13]. These examples highlight how single amino acid mutations at the virus-host interface can redefine which cells, tissues, or even species a virus can efficiently infect, often with major consequences for disease and One Health interventions.

Despite occurring in structurally and functionally distinct proteins, these mutations converge on a common principle where they change viral fitness by changing compatibility with different host proteins at critical interactions. Whether through enhanced polymerase activity, improved receptor binding, altered membrane fusion, or more efficient replication in specific cellular environments, these single amino acid changes act at inflection points within the viral life cycle. Notably, these amino acids involve changes in charge, polarity, or steric constraints that likely subtly reshape protein – protein interactions without globally disrupting structure. This recurring theme suggests that viral evolution often exploits a limited set of interaction points where minimal genetic change yields maximal phenotypic gain. As such, identifying and monitoring these residues provides not only insight into viral pathogenesis but also a predictive framework for anticipating emerging high-risk variants.

## Single amino acids can have global Health impact

The implications extend beyond pathogenesis to viral evolution. Mutations such as VP2 S447 represent more than incremental variation since they act as points of pathogenesis reprogramming. This small amino-acid level structural change redirect cellular targeting and reshape disease in a single step. This provides a mechanism for rapid shifts in virulence without the need for gradual accumulation of changes or large shifts in genomes. In this sense, structural mutations define not just phenotype, but evolutionary opportunity for the virus.

The observation that the S447 amdoparvovirus can infect human monocytic cell lines further sharpens this perspective. While no human disease has been attributed to amdoparvoviruses, the capacity to replicate in human immune cells strongly suggests evolutionary steps toward broader host adaptation have already been taken. The boundary between animal-restricted infection and cross-species potential may be narrower than assumed and defined by only a few critical residues.

This study also highlights a practical challenge. As seen during the COVID-19 pandemic, advances in sequencing have made it routine to catalog viral diversity, but sequence alone does not reveal function and cannot reliably predict public health impact without functional validation [14]. If single amino acid changes can drive major shifts in virulence, then identifying which mutations matter becomes essential. Functional validation and reverse genetics are key in interpreting risk associated with mutations in the viral genome.

In summary, Yang et al. provide a clear demonstration of a fundamental principle that viral virulence can be structurally encoded and rapidly reprogrammed at a single point in a virus. This important lesson is not limited to amdoparvoviruses as across virus families, the same logic applies. Small changes at the interface of virus and host cell proteins can produce large consequences for disease. Understanding how these changes occur and what they do remains central to predicting how viruses evolve and cause disease both at the cellular level and globally.

## Data Availability

Data sharing is not applicable to this article as no new data were created or analyzed in this study.

## References

[1] Mandl JN, Ahmed R, Barreiro L, et al. Reservoir host immune responses to emerging zoonotic viruses. Cell. 2015;160(1–2):20–4. doi: 10.1016/j.cell.2014.12.003 25533784 PMC4390999

[2] Yang L, Nie L, Ren H, et al. Isolation and characterization of a red panda amdoparvovirus causing fatal respiratory disease: identification of VP2-S447 as a key determinant of acute lung injury. Virulence. 2026;17(1):2658906. doi: 10.1080/21505594.2026.2658906 41979076 PMC13114132

[3] Lopez-Astacio RA, Adu OF, Lee H, et al. The structures and functions of parvovirus capsids and missing pieces: the viral DNA and its packaging, asymmetrical features, nonprotein components, and receptor or antibody binding and interactions. J Virol. 2023;97(7):e0016123. doi: 10.1128/jvi.00161-23 37367301 PMC10373561

[4] Parrish CR. Chapter 6: Parvoviridae. In: Knipe DM, Howley PM, editors. Fields virology. 7th ed. Philadelphia (PA): Wolters Kluwer; 2021. p. 172–196.

[5] Dworak LJ, Wolfinbarger JB, Bloom ME. Aleutian mink disease parvovirus infection of K562 cells is antibody-dependent and is mediated via an Fc(gamma)RII receptor. Arch Virol. 1997;142(2):363–373. doi: 10.1007/s007050050082 9125049

[6] Chang SF, Sgro JY, Parrish CR. Multiple amino acids in the capsid structure of canine parvovirus coordinately determine the canine host range and specific antigenic and hemagglutination properties. J Virol. 1992;66(12):6858–6867. doi: 10.1128/jvi.66.12.6858-6867.1992 1331498 PMC240290

[7] Steel J, Lowen AC, Mubareka S, et al. Transmission of influenza virus in a mammalian host is increased by PB2 amino acids 627K or 627E/701N. PLOS Pathog. 2009;5(1):e1000252. doi: 10.1371/journal.ppat.1000252 19119420 PMC2603332

[8] Hatta M, Gao P, Halfmann P, et al. Molecular basis for high virulence of Hong Kong H5N1 influenza A viruses. Science. 2001;293(5536):1840–1842. doi: 10.1126/science.1062882 11546875

[9] Urbanowicz RA, McClure CP, Sakuntabhai A, et al. Human adaptation of Ebola virus during the West African outbreak. Cell. 2016;167(4):1079–1087.e5. doi: 10.1016/j.cell.2016.10.013 27814505 PMC5101188

[10] Diehl WE, Lin AE, Grubaugh ND, et al. Ebola virus glycoprotein with increased infectivity dominated the 2013–2016 epidemic. Cell. 2016;167(4):1088–1098.e6. doi: 10.1016/j.cell.2016.10.014 27814506 PMC5115602

[11] Tsetsarkin KA, Vanlandingham DL, McGee CE, et al. A single mutation in chikungunya virus affects vector specificity and epidemic potential. PLOS Pathog. 2007;3(12):e201. doi: 10.1371/journal.ppat.0030201 18069894 PMC2134949

[12] Vazeille M, Moutailler S, Coudrier D, et al. Two Chikungunya isolates from the outbreak of La Reunion (Indian Ocean) exhibit different patterns of infection in the mosquito, Aedes albopictus. PLOS ONE. 2007;2(11):e1168. doi: 10.1371/journal.pone.0001168 18000540 PMC2064959

[13] Yuan L, Huang X-Y, Liu Z-Y, et al. A single mutation in the prM protein of Zika virus contributes to fetal microcephaly. Science. 2017;358(6365):933–936. doi: 10.1126/science.aam7120 28971967

[14] Harvey WT, Carabelli AM, Jackson B, et al. SARS-CoV-2 variants, spike mutations and immune escape. Nat Rev Microbiol. 2021;19(7):409–424. doi: 10.1038/s41579-021-00573-0 34075212 PMC8167834

